# Transcriptomics and Metabolomics Reveal Mechanisms Underlying the Adaptation of *Lamiophlomis rotata* to High Altitudes

**DOI:** 10.3390/biology14111554

**Published:** 2025-11-05

**Authors:** Yunzhang Xu, Sangjie Jiancuo, Xiao Luo, Yu-E Ma, Xin Wu, Zhenzhong Wu, Hengxia Yin, Shaoshan Zhang, Wenbing Li, Huachun Sheng

**Affiliations:** 1 State Key Laboratory of Plateau Ecology and Agriculture, Qinghai University, Xining 810016, China; 2023990008@qhu.edu.cn (Z.W.); hengxiayin@qhu.edu.cn (H.Y.); 2 College of Agriculture and Animal Husbandry, Qinghai University, Xining 810016, China; 19899775339@163.com (S.J.); 15509723940@163.com (X.W.); 3 NMPA Key Laboratory for Quality Monitoring and Evaluation of Traditional Chinese Medicine, Chengdu Institute for Drug Control, Chengdu 610045, China; lx76209608@163.com; 4 Xi’an Agricultural Product Quality and Safety Inspection and Monitoring Center, Xi’an 710077, China; jieyougongzhu1314@163.com (Y.-E.M.); 80300101@swun.edu.cn (S.Z.); 5 Sichuan Provincial Qiang-Yi Medicinal Resources Protection and Utilization Technology and Engineering Laboratory, Southwest Minzu University, Chengdu 610225, China; 26600005@swun.edu.cn; 6 Tibetan Plateau Ethnic Medicinal Resources Protection and Utilization Key Laboratory of National Ethnic Affairs Commission of the People’s Republic of China, Southwest Minzu University, Chengdu 610225, China

**Keywords:** *Lamiophlomis rotata*, high altitude, amino acid metabolism, hydrogen sulfide, cell wall remodeling

## Abstract

*Lamiophlomis rotata* (Benth.) Kudo is a typical alpine medicinal plant. However, the mechanism underlying its adaptation to high altitudes remains incompletely understood. In this study, we integrated transcriptome and metabolome analyses. Specifically, we used third-generation sequencing for building a reference transcriptome and second-generation sequencing for differential gene expression analysis. Our findings revealed that the activation of the hydrogen sulfide signaling pathway and the reprogramming of amino acid metabolism are probable adaptation mechanisms. Different from previous reports, the hydrogen sulfide signaling may regulate the activity of cellulose synthase in addition to enhancement of antioxidant capacity and accumulation of osmolytes. By altering the agronomic traits of plants in a cell wall remodeling-dependent manner, it enables *L. rotata* to adapt to alpine stress. The accumulated amino acids not only store energy-efficient organic nitrogen as precursors for the synthesis of secondary metabolites but also act as signaling molecules to activate defense responses. Additionally, we propose a potential link between the hydrogen sulfide signaling pathway and amino acid metabolism. Overall, this study systematically explores the adaptation mechanism of *L. rotata* to high-altitude environments, offering a novel perspective for understanding the growth, development, stress responses, and secondary metabolic processes of alpine plants.

## Simple Summary

*Lamiophlomis rotata* is a perennial herb belonging to the *Lamiaceae* family and is widely distributed across the high-altitude regions of the Himalayas and the Qinghai–Tibet Plateau. As a traditional alpine medicinal plant, it has been used in ethnomedicine for its anti-inflammatory and analgesic properties, yet the molecular mechanisms underlying its adaptation to extreme environments have remained poorly understood. To address this gap, the present study employs a multi-omics approach by integrating PacBio full-length transcriptome sequencing, Illumina-based second-generation transcriptome data, and comprehensive metabolome profiling. These findings offer novel insights into alpine plant biology and may inform strategies for improving crop adaptability in changing climates.

## 1. Introduction

*Lamiophlomis rotata* (Benth.) Kudo, also called Tibetan Duyiwei, is a perennial herbaceous plant taxonomically classified under the genus *Lamiophlomis* within the subfamily *Lamioideae* of the *Lamiaceae* family. It is also a traditional Chinese medicinal herb with a profound historical legacy, the earliest documented record of whose use dates back more than 1300 years [1]. To date, a substantial body of phytochemical research has resulted in the isolation and structural characterization of approximately 223 distinct chemical constituents from various anatomical parts of *L. rotata*, including leaves, stems, and roots [1,2]. These bioactive compounds are systematically classified into several major chemical categories: the monoterpenoid compounds of iridoids, the polyphenolic compounds of flavonoids, phenylethanoid glycosides, polysaccharides, and organic acids [2,3,4]. These chemical constituents exhibit a diverse range of pharmacological properties, including analgesic, hemostatic, anti-inflammatory, antineoplastic, immunomodulatory, antioxidant, and cardioprotective activities [5,6,7,8]. 

*L. rotata* is predominantly found in China, Nepal, Bhutan, and the Indian state of Sikkim, with an altitudinal distribution ranging from 2700 to 4500 m above sea level [1,2]. Its native habitat has features like low temperature, low oxygen, strong ultraviolet rays, and a big gap between day and night temperatures [9,10]. As a typical alpine species, *L. rotata* predominantly exhibits a prostrate growth habit close to the ground, and its low stature and well-developed rhizomes represent adaptive traits that enable the plant to survive in the harsh ecological conditions [11]. Recent transcriptomic results suggest that in the low-altitude *L. rotata*, the differentially expressed genes are primarily associated with growth-related functions, whereas in the high-altitude samples, they show a greater inclination towards defense responses [12]. In a previous study, a comparative proteomics analysis of *L. rotata* plants cultivated under three distinct altitude conditions demonstrated that high-altitude environments significantly upregulated the expression levels and enzymatic activities of proteins involved in hydrogen sulfide (H_2_S) biosynthesis, thereby promoting H_2_S accumulation [13]. Elevated H_2_S levels enhanced antioxidant enzyme activity, reduced oxidative damage caused by reactive oxygen species (ROS) and reactive nitrogen species (RNS), and induced protein degradation, along with the accumulation of proline and soluble sugars [13]. This study has provided initial insights into the mechanisms underlying the adaptation of *L. rotata* to high-altitude environments through integrative analyses of plant physiology and proteomics. Nevertheless, the molecular processes by which high-altitude stress triggers H_2_S signaling, reprograms the transcriptomic landscape, and modulates metabolic profiles remain largely unexplored.

Moreover, the mechanisms by which plants adapt to high-altitude environments are extremely intricate. To gain in-depth insights into the adaptation mechanisms of *L. rotata*, we performed comparative transcriptomic and metabolomic analyses on *L. rotata* plants grown under three different altitude conditions. The transcriptomic results revealed that, apart from enhancing antioxidant capacity, H_2_S-mediated cellulose synthesis may be an important high-altitude adaptation mechanism. Both transcriptomic and metabolomic findings indicated that high altitude influenced the accumulation of amino acids, suggesting that amino acid metabolism is also a probable mechanism for high-altitude adaptation.

## 2. Materials and Methods

### 2.1. Plant Material Collection and RNA Sample Preparation

**Figure 1:**
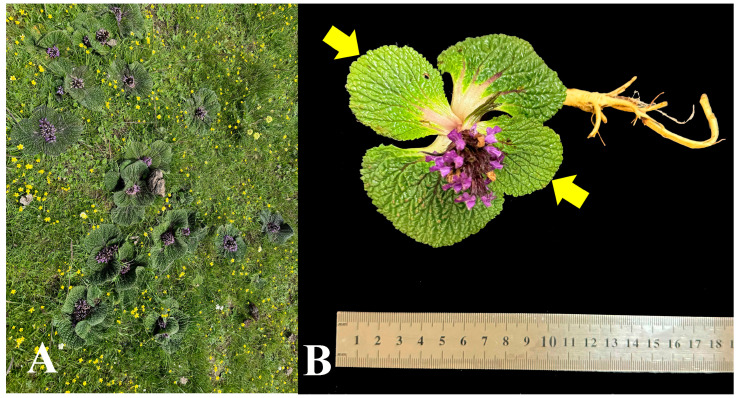
Habitat of *L. rotata* (**A**) and sampling parts (**B**). The yellow arrow indicates the sampling location.

To systematically investigate the variations in gene expression and metabolite accumulation along an altitude gradient, and based on the findings of preliminary field surveys, we selected three representative sampling sites at different altitudes in Zhongda Township, Yushu City, Qinghai Province (ranging from 3500 to 4600 m above sea level). Sampling was conducted in August 2023 at the following geographical coordinates: 33°15′58″ N, 96°54′23″ E (elevation: 3500 m), 33°18′6″ N, 96°58′50″ E (elevation: 4000 m), and 33°17′14″ N, 96°57′51″ E (elevation: 4665 m). Specimens of *L. rotata* were collected at each of these sites. To minimize potential influences of weather conditions, sampling was carried out exclusively on clear days between 11:00 AM and 4:00 PM. Plants exhibiting healthy growth and at the full-bloom developmental stage were selected. The third to fourth leaves measured from the base of the root were collected from each individual plant, with petioles deliberately excluded (Figure 1). At each sampling site, four individual plants were collected within a 10 m^2^ area. All samples were immediately flash-frozen in liquid nitrogen and stored at −80 °C for subsequent molecular analysis. Total RNA was extracted using the Trizol reagent kit (Invitrogen, Carlsbad, CA, USA) following the manufacturer’s instructions. RNA integrity was assessed using an Agilent 2100 Bioanalyzer (Santa Clara, CA, USA) and agarose gel electrophoresis, while RNA purity and concentration were determined using a NanoDrop micro-spectrophotometer (Thermo Fisher Scientific, Waltham, MA, USA).

### 2.2. PacBio Single-Molecule Real-Time (SMRT) Sequencing Library Preparation and Sequencing

Total RNA was isolated from leaves collected at three altitude gradients (3500 m, 4000 m, and 4600 m) and pooled in equal quantities to generate a composite RNA sample representing *L. rotata*. mRNA was enriched using Oligo(dT) magnetic beads, followed by reverse transcription into cDNA using the SMARTer^®^ PCR cDNA Synthesis Kit (TOYOBO, Tokyo, Japan). Subsequently, RNA fragments larger than 5 kb were size-selected using the BluePippin™ Size-Selection System and combined in equal proportions with the non-size-selected cDNA. Large-scale PCR amplification was then carried out in preparation for SMRTbell library construction. The cDNA was subjected to DNA damage repair, end-repair, and adapter ligation. The resulting SMRTbell template library was annealed with the sequencing primer, bound to the polymerase enzyme, and sequenced on the PacBio Sequel II platform (Gene Denovo Biotechnology Co., Guangzhou, China).

### 2.3. Bioinformatics Analysis for Isoform Sequencing

The raw sequencing reads from the cDNA library were analyzed using the Iso-Seq pipeline provided by Pacific Biosciences [14]. Initially, high-quality circular consensus sequences (CCS) were extracted from the subread BAM files. Transcript integrity was assessed by the presence of the 5′ primer, 3′ primer, and polyA tail within each CCS read. Sequences containing all three features were categorized as full-length (FL) reads. Following this, primers, barcodes, and polyA tails were removed, and tandem repeat regions within the FL reads were processed to generate full-length non-chimeric (FLNC) reads. These FLNC reads were subsequently clustered to identify distinct transcript isoforms. Hierarchical clustering of similar FLNC reads was performed using Minimap2 software v2.26 [15], resulting in uncorrected consensus isoforms. These consensus sequences were further refined using the Quiver algorithm to improve sequence accuracy. Additionally, low-quality transcript isoforms were corrected using the LoRDEC tool (https://www.lirmm.fr/~rivals/lordec/ (accessed on 18 May 2025)) in combination with filtered Illumina RNA-Seq data. High-quality transcript isoforms with a predicted accuracy of ≥0.99 were selected for downstream analysis.

To annotate transcript isoforms, the BLASTx program (http://www.ncbi.nlm.nih.gov/BLAST/ (accessed on 18 May 2025)) was applied to perform sequence similarity searches against the NCBI non-redundant protein (Nr) database (http://www.ncbi.nlm.nih.gov (accessed on 18 May 2025)), the Swiss-Prot protein database (http://www.expasy.ch/sprot (accessed on 18 May 2025)), the Kyoto Encyclopedia of Genes and Genomes (KEGG) database (http://www.genome.jp/kegg (accessed on 18 May 2025)), and the COG/KOG database (http://www.ncbi.nlm.nih.gov/COG (accessed on 18 May 2025)). An E-value threshold of 1 × 10^−5^ was used to identify significant matches with genes from other species. Gene Ontology (GO) annotation was conducted using Blast2GO software v1.3.3 [16], based on the Nr annotation results. Only isoforms with a top 20 alignment score and at least 33 high-scoring segment pairs (HSPs) were selected for GO annotation analysis. Functional classification of annotated isoforms was subsequently performed using WEGO software v2.0 [17]. Open reading frames (ORFs) were identified in the isoform sequences using ANGEL software v.3.0 [18], enabling the extraction of coding sequences (CDS), corresponding protein sequences, and untranslated regions (UTRs). Protein-coding sequences were further analyzed by aligning them to the Plant TFdb database (http://planttfdb.cbi.pku.edu.cn/ (accessed on 18 May 2025)) using hmmscan to predict transcription factor families. Additionally, BLASTp was employed to align these sequences against the PRGdb R-gene database (https://www.prgdb.org/prgdb4/ (accessed on 18 May 2025)) for the identification of potential R genes. The protein-coding potential of unannotated transcripts was evaluated using CNCI (version 2) [19] under default parameters to identify putative long non-coding RNAs (lncRNAs). For evolutionary annotation of lncRNAs, sequence alignment was performed using Infernal software v1.1.5 [20] (http://eddylab.org/infernal/ (accessed on 18 May 2025)), and lncRNAs were classified based on their secondary structures and sequence conservation. To systematically investigate alternative splicing events among transcript isoforms, the Coding Genome Reconstruction Tool (Cogent) was first used to group transcripts into distinct gene families according to k-mer similarity. Each gene family was then reconstructed into a representative coding reference genome using the De Bruijn graph algorithm. Finally, the SUPPA tool was employed to analyze and classify alternative splicing events within the isoforms.

### 2.4. Illumina RNA-Seq Library Preparation and Sequencing

RNA samples collected from three distinct altitude gradients were utilized for Illumina library construction and high-throughput sequencing. The workflow for constructing cDNA libraries for Illumina HiSeq™ 4000 sequencing was as follows: Total RNA was first subjected to mRNA enrichment using oligo(dT) magnetic beads. The enriched mRNA was subsequently fragmented into short fragments of approximately 200–300 nucleotides using fragmentation buffer. First-strand cDNA synthesis was initiated using random hexamer primers, followed by second-strand cDNA synthesis, which was performed with DNA polymerase I, RNase H, deoxyribonucleoside triphosphates (dNTPs), and reaction buffer. The resulting double-stranded cDNA fragments were purified using the QIAquick PCR Purification Kit (Qiagen, Venlo, Netherlands). The purified fragments were then subjected to end repair, poly(A) tail addition, and ligation with Illumina TruSeq adapters in a sequential manner. Following adapter ligation, the products were size-selected via agarose gel electrophoresis and subsequently amplified by PCR. The final libraries were sequenced on the Illumina HiSeq™ 4000 platform at Guangzhou GENE Denovo Biotechnology Co., Ltd. (Guangzhou, China). Following RNA sequencing, raw reads were processed to generate high-quality clean reads through a series of filtering steps. These included the removal of adapter sequences, elimination of reads containing more than 10% unknown nucleotides (N), and filtering of low-quality reads. After quality trimming and filtering, the Q30 score and GC content of the clean reads were calculated for further quality assessment.

### 2.5. Transcript Abundance and Differential Expression Analyses

Using the full-length transcripts (isoforms) obtained from third-generation sequencing as reference sequences, quantitative analysis was performed on the second-generation transcriptome data of *L. rotata* leaves sampled across three altitudinal gradients. Gene expression abundance and variation were calculated using RSEM software v1.3.112 and normalized to FPKM (fragments per kilobase of transcript per million mapped reads). RNA differential expression analysis between two distinct sample groups was conducted using DESeq2, while edgeR was employed for pairwise comparisons between individual samples. Genes satisfying the thresholds of |log_2_FC| > 1.5, *p* < 0.05, and FDR < 0.05 were identified as differentially expressed genes (DEGs). Subsequently, Gene Ontology (GO) and Kyoto Encyclopedia of Genes and Genomes (KEGG) enrichment analyses were carried out to explore the functional implications of the identified DEGs.

### 2.6. Metabolite Extraction and Sample Preparation

To identify metabolites with significant biological relevance, a widely targeted metabolomics approach was employed to characterize the composition and quantitative profiles of metabolites in *L. rotata* across varying altitudinal conditions. The experimental procedure for the liquid chromatography-mass spectrometry (LC-MS)-based widely targeted metabolomics analysis was as follows [21]: 100 mg of sample material was transferred into a 2.0 mL microcentrifuge tube, followed by the addition of 500 μL of 80% methanol–water solution. The mixture was vortexed thoroughly, incubated in an ice bath for 5 min, and subsequently centrifuged at 15,000× *g* for 20 min at 4 °C. An aliquot of the resulting supernatant was diluted with water to achieve a final methanol concentration of 53%, followed by a second centrifugation step under the same conditions (15,000× *g*, 20 min, 4 °C) to collect the clarified supernatant. For each sample, four biological replicate samples were prepared. Subsequently, these samples were submitted to Guangzhou Genedenovo Biotechnology Co., Ltd. (Guangzhou, China) for analysis via LC-MS.

### 2.7. LC-MS Metabolomic Analysis

Metabolite profiling was carried out using a SCIEX liquid chromatography–mass spectrometry system (SCIEX, Framingham, MA, USA) coupled with an Xselect HSS T3 column (2.1 × 150 mm, 2.5 μm) maintained at 50 °C [21]. The system was operated in both electrospray ionization (ESI) positive and negative ion modes. Mobile phase A consisted of 0.1% formic acid in water, while mobile phase B contained 0.1% formic acid in acetonitrile. The gradient elution program was set as follows: 0–14.9 min, 98% A; 15–17 min, 0% A; and 17.1–20 min, 98% A. The injection volume was 2 μL, with a flow rate of 0.4 mL/min.

Samples were analyzed using the multiple reaction monitoring (MRM) mode, based on the metabolite database established by Guangzhou Genedenovo Biotechnology Co., Ltd. Quantitative analysis was performed using Q3 (product ions), whereas qualitative identification relied on Q1 (precursor ions), Q3 (product ions), retention time (RT), declustering potential (DP), and collision energy (CE). Raw mass spectrometry data were processed using SCIEX OS V1.4 software, which enabled chromatographic peak integration, baseline correction, and peak filtering according to predefined parameters: minimum peak height of 500, signal-to-noise ratio of 5, and smoothing over 1 point. The peak area of each chromatographic peak was used as an indicator of the relative abundance of the corresponding metabolite. All integrated peak area data were exported for subsequent qualitative and quantitative metabolite analysis.

The LC–MS data matrix was imported into R software v 4.5.1 for multivariate statistical analysis. Principal component analysis (PCA) was performed to evaluate the overall stability and distribution of samples during the analytical process. Additionally, (orthogonal) partial least squares discriminant analysis ((O)PLS-DA) was applied to identify global metabolic differences between experimental groups. Variable importance in projection (VIP) values were calculated based on the OPLS-DA model, with metabolites exhibiting VIP > 1 considered as potential differential metabolites (DAMs). These candidates were further subjected to *t*-tests to assess the statistical significance of intergroup differences (*p* < 0.05).

## 3. Results

### 3.1. General Characteristics and Functional Annotation of the Full-Length Transcriptome in Leaves of L. rotata

**Figure 2:**
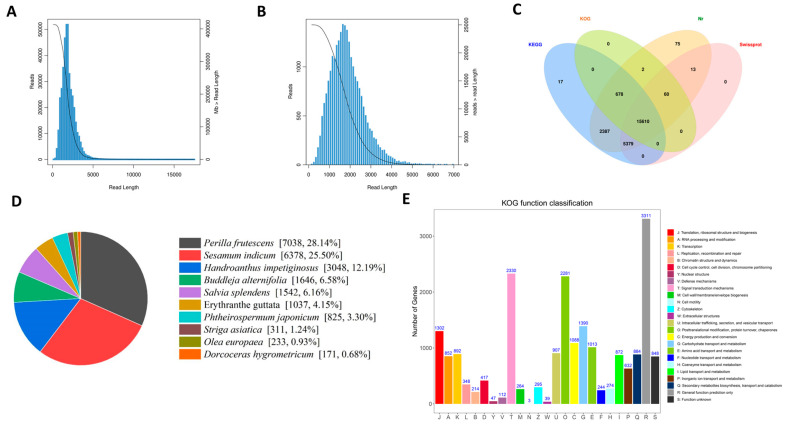
Summary of full-length transcriptome sequences. (**A**) Length distribution of CCS reads. (**B**) Distribution of high-quality isoform lengths. (**C**) Venn diagram of annotation results from four major databases. (**D**) Homologous species distribution of *L. rotata* based on the NR database. (**E**) Statistics of KOG annotation information.

We constructed a full-length transcriptome library using RNA extracted from leaves of *L. rotata* collected along different elevation gradients. SMRT sequencing produced 99.0 GB of raw sequencing data, resulting in 412,983 high-quality circular consensus sequences (CCSs), with an average length of 1967 bp and an N50 of 2114 bp (Table S1; Figure 2A). Following the removal of adapters and low-quality sequences, a total of 336,585 full-length non-chimeric (FLNC) reads were obtained, representing 81.50% of the CCSs. The FLNC reads had an average length of 1801 bp, an N50 of 1961 bp, Q20 and Q30 scores of 93.81% and 90.79%, respectively, and a GC content of 46.12% (Table S1).

Using Minimap2, similar FLNC reads were hierarchically clustered, yielding 31,976 high-quality isoforms and 107 low-quality isoforms (Table S2). Following this, polishing with the Quiver algorithm, redundancy removal via cd-hit-v4.6.7, and the elimination of low-quality sequences resulted in a total of 25,012 refined isoforms. These isoforms ranged in length from 145 to 7000 bp and were distributed across the following size intervals: 268 (0–0.5 kb), 2969 (0.5–1 kb), 12,427 (1–2 kb), 7178 (2–3 kb), and 2170 (>3 kb). The average length was 1857.67 bp, with an N50 of 2081 bp (Table S1; Figure 2B). 

For functional annotation, the 25,012 non-redundant isoforms were subjected to BLASTx analysis against the Nr, SwissProt, KEGG, and COG/KOG protein databases. A total of 24,221 isoforms were successfully annotated, of which 15,610 were annotated across all four databases. Specifically, 24,204 and 21,062 isoforms were annotated in the Nr and SwissProt databases, respectively (Table S3; Figure 2C). To evaluate sequence conservation, the Nr-annotated isoforms were compared with sequences from other plant species, revealing the highest homology with *Perilla frutescens* (28.14%), followed by *Sesamum indicum* (25.50%), and *Handroanthus impetiginosus* (12.19%) (Figure 2D).

Additionally, 20,859 full-length transcripts were annotated in the KOG database, with the largest category being “General function prediction only,” comprising 3311 isoforms (Figure 2E). Gene Ontology (GO) annotation assigned 25,003 isoforms to three main categories: biological processes (66,469 terms), molecular functions (33,829 terms), and cellular components (18,070 terms) (Figure S1). KEGG pathway analysis revealed that 7992 isoforms were mapped to 139 subcategories within five major functional categories (Table S4; Figure S2). The most enriched pathways were “Metabolic pathways” (ko01100; 4595 isoforms) and “Biosynthesis of secondary metabolites” (ko01110; 2569 isoforms).

### 3.2. Evaluation of RNA-Seq Data and Identification of Ddifferentially Expressed Genes in Leaves of L. rotata Across Altitudinal Gradients

**Figure 3:**
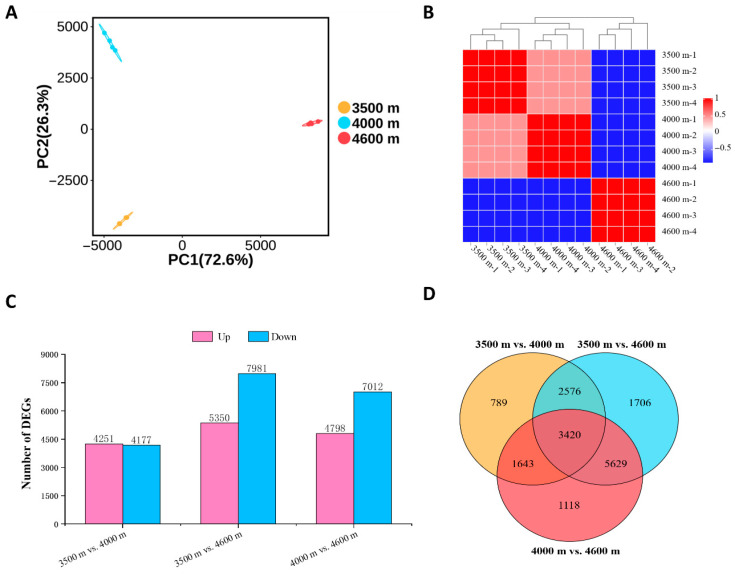
Quality control analysis of RNA-Seq data and differentially expressed genes (DEGs) in comparison group. (**A**) Principal component analysis (PCA) of all the samples. (**B**) The heatmap of Pearson’s correlation of 12 samples. (**C**) The number of upregulated (purple) and downregulated (blue) DEGs. (**D**) The interrelationships of DEGs among different comparison groups.

The PacBio-assembled isoforms were utilized as the reference transcriptome for aligning Illumina short reads, and gene expression profiles were subsequently generated using FPKM normalization across 12 samples collected along three elevation gradients (four biological replicates per group). RNA-seq analysis yielded 39,714,232 to 71,367,020 clean reads per sample, with Q30 scores ranging from 92.80% to 94.17%. Mapping analysis indicated overall alignment rates between 80.59% and 84.07%, comprising 17.66% to 18.65% uniquely mapped reads and 62.12% to 66.31% multi-mapped reads (Table S5). Principal component analysis (PCA) revealed distinct separation among elevation groups (Figure 3A), while high correlation among biological replicates (Figure 3B) confirmed the reliability of the dataset for downstream differential expression analysis.

To identify candidate genes potentially involved in altitude adaptation, pairwise comparisons of gene expression profiles were conducted across three elevation gradients. The analysis identified 13,331 differentially expressed genes (DEGs) between 3500 m and 4600 m (5350 up-regulated and 7981 down-regulated), 11,810 DEGs between 4000 m and 4600 m (4798 up-regulated and 7012 down-regulated), and 8428 DEGs between 3500 m and 4000 m (4251 up-regulated and 4,177 down-regulated). Notably, the two comparisons involving the highest elevation (4600 m) exhibited a significantly higher number of down-regulated than up-regulated genes (Figure 3C), suggesting that transcriptional suppression may predominate during the adaptation process. Moreover, 3420 commonly shared DEGs were identified across all comparisons (Figure 3D), which likely represent core regulatory components associated with altitudinal adaptation.

### 3.3. DEGs Are Predominantly Associated with Sulfur Metabolism, Cell Wall Remodeling Processes, and Amino Acid Metabolic Activities

**Figure 4:**
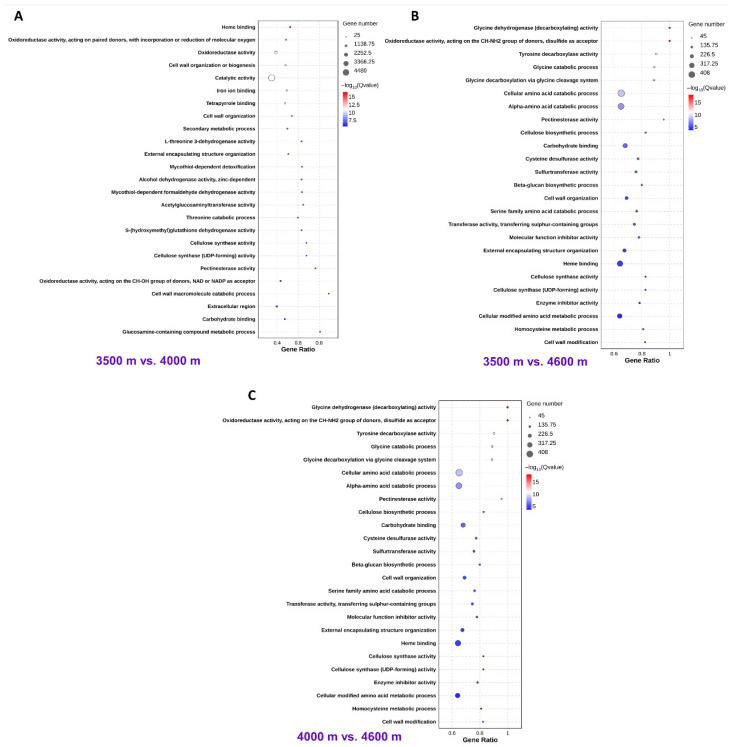
Comparative analysis of GO enrichment. GO analysis of DEGs in 3500 m vs. 4000 m (**A**), 3500 m vs. 4600 m (**B**), and 4000 m vs. 4600 m (**C**).

To investigate the molecular mechanism underlying the adaptation of *L. rotata* to high altitudes, we performed Gene Ontology (GO) enrichment analysis on the DEGs identified in the three comparison groups (3500 m vs. 4000 m, 3500 m vs. 4600 m, and 4000 m vs. 4600 m). GO enrichment analysis reveals that DEGs are predominantly associated with sulfur metabolism, cell wall remodeling processes, and amino acid metabolic activities (Figure 4). Specifically, the sulfur metabolism-related DEGs that were functionally enriched in oxidoreductase, cysteine desulfurase, and sulfur metabolic processes (such as sulfur-transferase activity) may be involved in the biosynthesis of endogenous H_2_S in plants. The reason is that cysteine desulfurase can utilize L-Cys as a substrate in the presence of pyridoxal 5′-phosphate (PLP) to produce L-alanine and elemental sulfur; if a reducing agent (electron donor) is present in the working environment of this enzyme, elemental sulfur will be reduced to form H_2_S. It is evident that DEGs enriched in biological processes such as cell wall organization or biogenesis, cellulose synthase activity, and pectinesterase activity are closely associated with cell wall remodeling. Similarly, DEGs enriched in metabolic pathways including glycine catabolism, tyrosine decarboxylation, serine family amino acid catabolism, and general cellular amino acid catabolism are implicated in amino acid metabolic activities.

### 3.4. Metabolomics Analysis and Identification of Differential Metabolites

**Figure 5:**
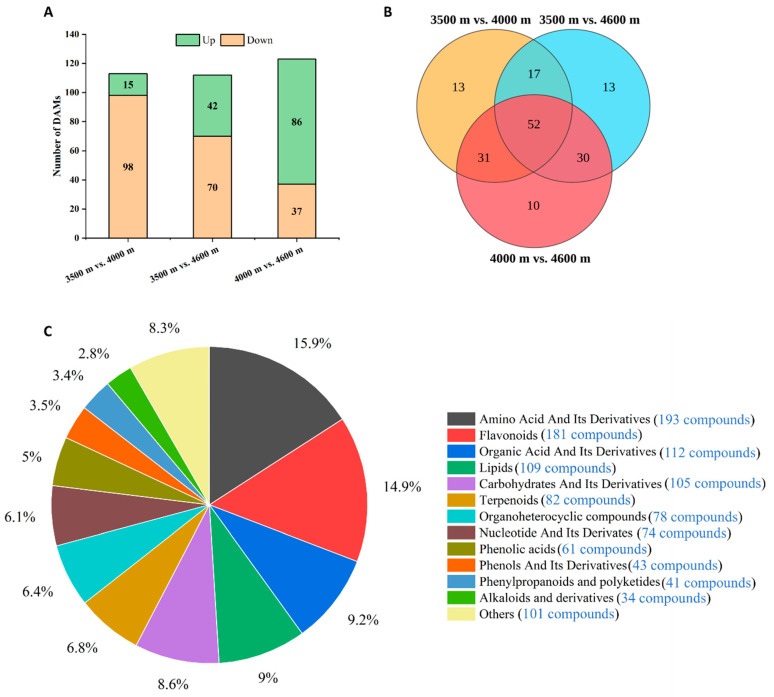
Metabolomics analysis of differentially accumulated metabolites (DAMs). (**A**) The quantities of DAMs in three comparison groups. (**B**) The interrelationships of DAMs among three comparison groups. (**C**) Chemical classification of DAMs, along with the quantities and percentages of each type of differential metabolite.

KEGG pathway analysis of DEGs demonstrated that elevated altitude not only activated amino acid metabolism but also modulated the biosynthesis of other metabolites (Figure S3). To corroborate the transcriptomic findings, metabolomics approaches were applied to systematically investigate the variations in metabolite accumulation. The principal component analysis (PCA) and the heatmap of Pearson correlation of all samples demonstrate the reliability of the metabolomic data (Figure S4). In the three comparison groups (3500 m vs. 4000 m, 3500 m vs. 4600 m, and 4000 m vs. 4600 m), the numbers of metabolites exhibiting increased accumulation were 15, 42, and 86, respectively, whereas the numbers of metabolites showing decreased accumulation were 98, 70, and 37, respectively (Figure 5A). Among these differentially accumulated metabolites (DAMs), 52 were common across all three comparison groups (Figure 5B). Specifically, 13 DAMs were uniquely identified in both the 3500 m vs. 4000 m and 3500 m vs. 4600 m comparison groups, whereas 10 were exclusively observed in the 4000 m vs. 4600 m comparison group (Figure 5B). Compound classification and quantitative statistical analysis revealed that amino acids and their derivatives, flavonoids, and organic acids and their derivatives represented the three most abundant categories among the DAMs, accounting for 15.9% (193 compounds), 14.9% (181 compounds), and 9.2% (112 compounds), respectively (Figure 5C).

### 3.5. The Accumulation of the Majority of Amino Acids and Their Derivatives Among the Differential Metabolites Increases as the Altitude Ascends

**Figure 6:**
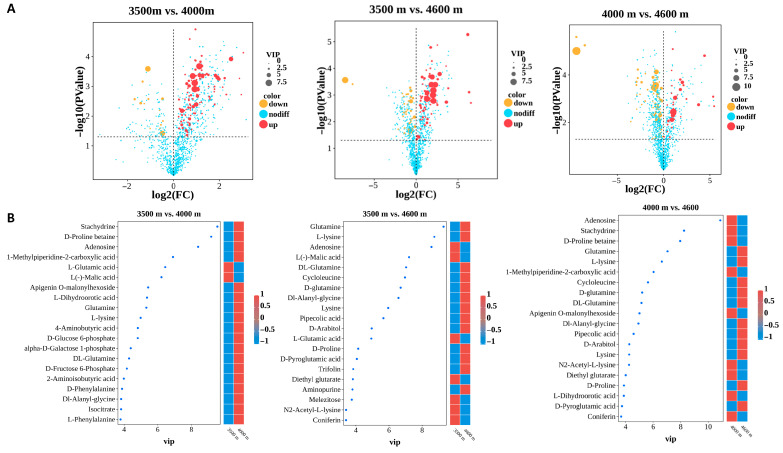
Analysis and identification of important differential metabolites. (**A**) Volcano plot reveals potential important differential metabolites. (**B**) The top 20 identified important differential metabolites.

**Figure 7:**
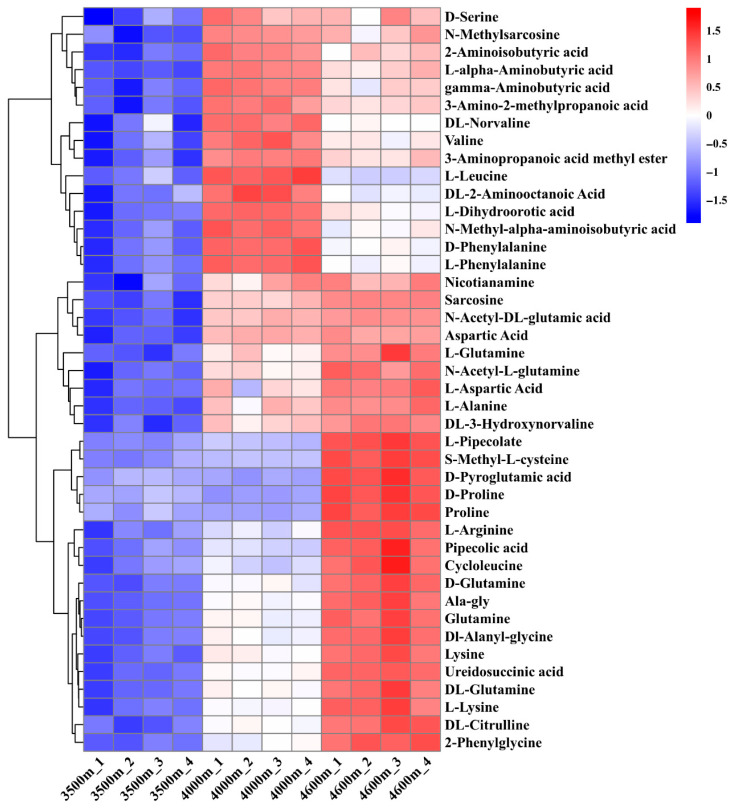
The relationship between the accumulation amounts of amino acids and their derivatives and altitude. The data indicate that the accumulation levels of the majority of amino acids and their derivatives exhibit an upward trend as altitude increases.

We performed a comprehensive analysis of differential metabolites through basic differential analysis (Figure 6A) combined with the calculation of variable importance in projection (VIP) values (Figure 6B). The results indicated that the majority of the top 20 most significant differential metabolites were amino acids and their derivatives. Among these, the amino acid-derived metabolites stachydrine (also known as proline betaine), adenosine, and D-proline betaine were ranked within the top three in both comparison groups (3500 m vs. 4000 m and 4000 m vs. 4600 m) (Figure 6B). In the 3500 m vs. 4600 m comparison group, glutamine, L-lysine, and adenosine were identified as the top three differential metabolites (Figure 6B). Given that stachydrine, adenosine, and D-proline betaine differ significantly in chemical structure from canonical amino acids, a systematic screening and analysis of differential accumulation patterns of amino acids and their derivatives among the identified metabolites were performed. The results revealed that most amino acids and their derivatives, including proline, arginine, and glutamine, displayed a consistent upward trend in accumulation with increasing altitude (Figure 7).

## 4. Discussion

High-altitude plants have evolved intricate mechanisms to adapt to different growth environments. Regarding *L. rotata*, previous proteomic studies have indicated that, as altitude increases, the content of endogenous H_2_S increases in plants, which leads to an enhancement of antioxidant capacity and the accumulation of osmoregulatory substances (proline and sugars), thereby improving its adaptability [13]. In this study, using multi-omics approaches such as full-length transcriptomics, second-generation transcriptomics, and metabolomics (Figure 2, Figure 3 and Figure 5), we systematically investigated the adaptation mechanism of *L. rotata*. Transcriptome analysis revealed that the DEGs induced by high altitude were predominantly associated with sulfur metabolism, cell wall remodeling, and amino acid metabolism (Figure 4). Among these, the discovery of sulfur metabolism-related DEGs may be associated with H_2_S signaling and further corroborates previous findings [13]. Meanwhile, the identification of DEGs related to cell wall remodeling and amino acid metabolism suggests the existence of other novel mechanisms for high-altitude adaptation in *L. rotata*.

The plant cell wall has cellulose microfibrils as its fundamental framework, within which hemicellulose, pectin, lignin, and structural proteins are embedded [22]. It not only provides mechanical support for plant growth, but also dictates the shape and size of plant cells [22]. Put another way, it determines the morphogenesis of plant organs and the phenotypic characteristics of plants [23,24]. The phenotypic traits of *L. rotata*, such as well-developed rhizomes, prostrate growth, and broad leaves, are adaptations to high-altitude environments (Figure 1). Consequently, that these traits are realized by modulating the synthesis and remodeling of the cell wall. Cellulose synthases (CESAs) are responsible for the biosynthesis of cellulose and represent a major component among the DEGs associated with cell wall remodeling (Figure 4). Previous studies have demonstrated that CESA-mediated cell wall synthesis and remodeling are of great significance for plants to respond to environmental stresses [25,26]. It has been reported that under stress conditions, the remodeling of guard cell walls in plants is crucial for optimizing photosynthetic efficiency and water use efficiency [27]. Interestingly, the exogenous application of H_2_S has been shown to enhance cellulose synthesis and modify the arrangement of cellulose microfibrils by increasing the rate of mobility of CESA complexes [28]. Therefore, apart from enhancing the antioxidant capacity and promoting the accumulation of osmoregulatory substances, H_2_S may improve the adaptability of *L. rotata* to high altitudes by regulating the synthesis and remodeling of the cell wall. Herein, we have not presented conclusive evidence to support this hypothesis, which calls for further investigation. This is primarily due to our greater focus on the role of amino acid metabolism in the adaptation of *L. rotata* to high-altitude environments.

Given that transcriptome analysis revealed numerous DEGs associated with amino acid metabolism, we employed metabolomic techniques to conduct a focused analysis of the differences in the contents of various amino acids in the leaves of *L. rotata* growing at different altitudes (Figure 5). Among these, the concentrations of proline, arginine, lysine, aspartic acid, and other amino acid derivatives (e.g., S-methyl-L-cysteine and glutamine) were found to increase with increasing altitude (Figure 7). Numerous studies have demonstrated that the accumulation of amino acids plays a crucial role in enabling plants to withstand abiotic stresses [29,30]. The proteinogenic amino acid of proline is commonly acknowledged to function as an osmoprotectant, a scavenger of reactive oxygen species, and a stabilizer of DNA, proteins, and cell membranes under the stressed conditions [29]. Numerous studies have indicated that plants exhibit increased proline levels when exposed to a range of abiotic stressors, such as drought, salinity, ultraviolet radiation, heavy metal contamination, and oxidative stress [31,32,33]. The accumulation of arginine can act as a precursor for the synthesis of various secondary metabolites, particularly nitrogen-containing compounds such as nitric oxide and polyamines, which are known to contribute significantly to abiotic stress tolerance [34,35]. Additionally, elevated levels of tryptophan enhance cold stress resistance and reduce the bioavailability of cadmium (Cd) in plants, likely due to interactions between metal ions and the divalent side chain of tryptophan [28]. Moreover, glycine, glutamic acid, and cysteine serve as essential precursors in the synthesis of glutathione, a key antioxidant in biological systems [36,37,38]. 

During the various stages of plant growth, development, and response to environmental stressors, amino acids and their derivatives have been found to contribute significantly to the activation of defense mechanisms within plants as well [39]. These organic compounds not only serve as building blocks for proteins but also function as signaling molecules that help plants adapt to changing conditions [40]. Among these amino acids, isoleucine and glutamine have garnered particular attention due to their demonstrated ability to activate the Target of Rapamycin (TOR) pathway [41,42]. This pathway is a highly conserved regulatory hub across eukaryotic organisms and plays a central role in integrating nutrient availability with cellular metabolism and growth-related signaling processes [43,44]. Meanwhile, the inhibition of TOR also leads to the extensive accumulation of amino acids, suggesting a feedback mechanism that may be linked to the plant’s adaptive response to nutrient imbalance or environmental stress [45,46]. For instance, TOR orchestrates the amino acid-derived metabolism to maintain a balance between plant growth and cold stress tolerance in tomato [47].

Furthermore, the accumulation of amino acids is considered to be one of the crucial adaptive strategies employed by alpine plants to cope with severe environmental conditions. Kumari et al. (2020) observed that the amino acid content in *Picrorhiza kurroa* exhibited organ-specific variations across different altitudes [48]. Specifically, the concentrations of histidine and arginine in the leaves rose significantly at elevations of 3800 and 4100 m above sea level, while proline levels showed an increase exclusively at the higher altitude of 4100 masl [48]. Similar results were also observed in *Herpetospermum pedunculosum* [49]. Moreover, as altitude increased, the levels of all amino acids across various metabolites were observed to be elevated in *Rhodiola crenulata* plants. The underlying mechanisms are: (1) accumulation of amino acids under alpine stress conditions may be linked to nitrogen storage and serve as a precursor for the synthesis of secondary metabolites; (2) distinct accumulation patterns of these amino acids under high altitude allow for more efficient energy use when amino acid biosynthesis and degradation occur simultaneously. Our findings are broadly consistent with previous studies, indicating that the mechanism by which *L. rotata* accumulates amino acids to adapt to high-altitude environments is comparable to those reported in earlier literature [48,49,50]. We also hypothesize that alterations in amino acid metabolism can initiate signal transduction pathways associated with high-altitude adaptation. This will be a focus of our future research endeavors.

Notably, H_2_S is synthesized from the amino acid cysteine through a series of enzymatic reactions, primarily catalyzed by cysteine desulphydrases (CDes) [51,52]. This process is also induced by high-altitude conditions in *L. rotata*, suggesting an inherent link between H_2_S and amino acid metabolism. Given that amino acids not only serve as precursors for H_2_S synthesis but also play key roles in osmoregulation, antioxidant defense, and protein synthesis, understanding the interplay between these metabolic pathways could provide valuable insights into stress tolerance mechanisms. Therefore, future studies should concentrate on elucidating the specific role of H_2_S signaling in modulating amino acid metabolic pathways under stress conditions.

## 5. Conclusions

In this study, transcriptomic and metabolomic approaches were systematically applied to investigate the molecular mechanisms underlying the adaptation of *L. rotata* to high-altitude environments. The results demonstrated that the H_2_S signaling pathway was markedly activated under high-altitude stress conditions, accompanied by significant alterations in cell wall remodeling. Moreover, amino acid metabolic profiles were reprogramed as well, particularly the accumulation of key stress-resistant amino acids such as proline, glutamine, and cysteine. These biochemical changes enhance plant tolerance to alpine stress through the modulations of plant morphology and relevant signaling pathways. Collectively, these findings suggest that the activation of H_2_S signaling and the reprogramming of amino acid metabolism represent crucial adaptive strategies in *L. rotata*, thereby providing a novel theoretical foundation for understanding the stress resistance mechanisms of alpine plant species.

## Data Availability

Additional supporting information may be found online in the Supplementary Materials section at the end of the article. The transcriptome data that support the findings of this study have been deposited into NGDC database (https://ngdc.cncb.ac.cn/gsub/) (accessed on 18 May 2025) with accession number CRA029276.

## Supplementary Materials

The following supporting information can be downloaded at: https://www.mdpi.com/article/10.3390/biology14111554/s1, Figure S1: Statistics of GO annotation information. Figure S2: Statistics of KEGG annotation information (Only display the first 53 pathways with the number of gene enrichments greater than 100). Figure S3: KEGG pathway analysis of DEGs demonstrated that elevated altitude not only activated amino acid metabolism but also modulated the biosynthesis of other metabolites. Figure S4: Quality control analysis of metabolomic data. (A) Principal component analysis (PCA) of all the samples. (B) The heatmap of Pearson’s correlation of 12 samples. Table S1: Summary of transcripts for *L. rotata*. Table S2: FLNC reads after clustering and error correction. Table S3: Summary of annotation results from four databases. Table S4: Genes for KEGG pathway analysis. Table S5: Information on RNA-seq clean reads mapped with the full-length transcripts.

## References

[1] Li Y., Li F., Zheng T.T., Shi L., Zhang Z.G., Niu T.M., Wang Q.Y., Zhao D.S., Zhao P. (2021). Lamiophlomis herba: A comprehensive overview of its chemical constituents, pharmacology, clinical applications, and quality control. Biomed. Pharmacother..

[2] Cui Z.H., Qin S.S., Zang E.H., Li C., Gao L., Li Q.C., Wang Y.L., Huang X.Z., Zhang Z.Y., Li M.H. (2020). Traditional uses, phytochemistry, pharmacology and toxicology of *Lamiophlomis rotata* (Benth.) Kudo: A review. RSC Adv..

[3] Liu J., Nan P., Wang L., Wang Q., Tsering T., Zhong Y. (2006). Chemical variation in lipophilic composition of *Lamiophlomis rotata* from the Qinghai-Tibetan Plateau. Chem. Nat. Compd..

[4] Yi J.H., Zhong C.C., Luo Z.Y., Xiao Z.Y. (1991). Studies on the chemical constituents from the roots of *Lamiophlomis rotata* (Benth.) Kudo, a medical plant in Xi-Zang (Tibet). Acta Pharm. Sin..

[5] Li M., Shang X., Zhang R., Jia Z., Fan P., Ying Q., Wei L. (2010). Antinociceptive and anti-inflammatory activities of iridoid glycosides extract of *Lamiophlomis rotata* (Benth.) Kudo. Fitoterapia.

[6] Fan P.C., Ma H.P., Hao Y., He X.R., Sun A.J., Jiang W., Li M.X., Jing L.L., He L., Ma J. (2016). A new anti-fibrinolytic hemostatic compound 8-O-acetyl shanzhiside methylester extracted from *Lamiophlomis rotata*. J. Ethnopharmacol..

[7] Zheng Y., Yin X., Huo F., Xiong H., Mei Z. (2015). Analgesic effects and possible mechanisms of iridoid glycosides from *Lamiophlomis rotata* (Benth.) Kudo in rats with spared nerve injury. J. Ethnopharmacol..

[8] Zhang D., Gao Y.L., Jiang S., Chen Y., Zhang Y., Pan Z. (2018). The similarity and variability of the iridoid glycoside profile and antioxidant capacity of aerial and underground parts of *Lamiophlomis rotata* according to UPLC-TOF-MS and multivariate analyses. RSC Adv..

[9] Zhang K.-L., Leng Y.-N., Hao R.-R., Zhang W.-Y., Li H.-F., Chen M.-X., Zhu F.-Y. (2024). Adaptation of high-altitude plants to harsh environments: Application of phenotypic-variation-related methods and multi-omics techniques. Int. J. Mol. Sci..

[10] Sun Y.B., Fu T.T., Jin J.Q., Murphy R.W., Hillis D.M., Zhang Y.P., Che J. (2018). Species groups distributed across elevational gradients reveal convergent and continuous genetic adaptation to high elevations. Proc Natl. Acad. Sci. USA.

[11] Sun P., Hao R., Fan F., Wang Y., Zhu F. (2025). Adaptation of high-altitude plants to plateau abiotic stresses: A case study of the Qinghai-Tibet plateau. Int. J. Mol. Sci..

[12] Wu X., Chen H., Ding R., Chen G., Jia H., Zhong S., Gu R. (2025). Integrating transcriptome, metabolome and microbiome to explore the molecular mechanism of phenotypic plasticity in *P. rotata* during low-altitude domestication. Plant Cell Environ..

[13] Ma L., Yang L., Zhao J., Wei J., Kong X., Wang C., Zhang X., Yang Y., Hu X. (2015). Comparative proteomic analysis reveals the role of hydrogen sulfide in the adaptation of the alpine plant *Lamiophlomis rotata* to altitude gradient in the Northern Tibetan Plateau. Planta.

[14] Gordon S.P., Tseng E., Salamov A., Zhang J., Meng X., Zhao Z., Kong D., Underwood J., Grigoriev I.V., Figueroa M. (2015). Widespread polycistronic transcripts in fungi revealed by single-molecule mRNA sequencing. PLoS ONE.

[15] Li H. (2018). Minimap2: Pairwise alignment for nucleotide sequences. Bioinformatics.

[16] Conesa A., Götz S., García-Gómez J.M., Terol J., Talón M., Robles M. (2005). Blast2GO: A universal tool for annotation, visualization and analysis in functional genomics research. Bioinformatics.

[17] Ye J., Fang L., Zheng H., Zhang Y., Chen J., Zhang Z., Wang J., Li S., Li R., Bolund L. (2006). WEGO: A web tool for plotting GO annotations. Nucleic Acids Res..

[18] Shimizu K., Adachi J., Muraoka Y. (2006). ANGLE: A sequencing errors resistant program for predicting protein coding regions in unfinished cDNA. J Bioinf. Comput. Biol..

[19] Sun L., Luo H., Bu D., Zhao G., Yu K., Zhang C., Liu Y., Chen R., Zhao Y. (2013). Utilizing sequence intrinsic composition to classify protein-coding and long non-coding transcripts. Nucleic Acids Res..

[20] Nawrocki E.P., Eddy S.R. (2013). Infernal 1.1: 100-fold faster RNA homology searches. Bioinformatics.

[21] Luo P., Dai W., Yin P., Zeng Z., Kong H., Zhou L., Wang X., Chen S., Lu X., Xu G. (2015). MRM-ion pair finder: A systematic approach to transform non-targeted mode to pseudo-targeted mode for metabolomics study based on liquid chromatography-mass spectrometry. Anal. Chem..

[22] Cosgrove D.J. (2024). Structure and growth of plant cell walls. Nat. Rev. Mol. Cell Biol..

[23] Bidhendi A.J., Geitmann A. (2016). Relating the mechanics of the primary plant cell wall to morphogenesis. J. Exp. Bot..

[24] Lamport D.T., Tan L., Held M., Kieliszewski M.J. (2018). The role of the primary cell wall in plant morphogenesis. Int. J. Mol. Sci..

[25] Li W., Wei J., Lei Y., Yang Z., Zhang S., Feng J., Li Y., Liu Y., Sheng H. (2025). Phosphorylation of cellulose synthases in plant responses to environmental changes. Int. J. Biol. Macromol..

[26] Kesten C., Menna A., Sánchez-Rodríguez C. (2017). Regulation of cellulose synthesis in response to stress. Curr. Opin. Plant Biol..

[27] Wang Y., Li P., Sun W., Zhang T. (2024). Plant cell walls: Emerging targets of stomata engineering to improve photosynthesis and water use efficiency. New Crops.

[28] Li J., Wang X., Wang X., Ma P., Yin W., Wang Y., Chen Y., Chen S., Jia H. (2020). Hydrogen sulfide promotes hypocotyl elongation via increasing cellulose content and changing the arrangement of cellulose fibrils in alfalfa. J. Exp. Bot..

[29] Batista-Silva W., Heinemann B., Rugen N., Nunes-Nesi A., Araújo W.L., Braun H.P., Hildebrandt T.M. (2019). The role of amino acid metabolism during abiotic stress release. Plant Cell Environ..

[30] Hildebrandt T.M. (2018). Synthesis versus degradation: Directions of amino acid metabolism during Arabidopsis abiotic stress response. Plant Mol. Biol..

[31] Krasensky J., Jonak C. (2012). Drought, salt, and temperature stress-induced metabolic rearrangements and regulatory networks. J. Exp. Bot..

[32] Saradhi P.P., Alia, Arora S., Prasad K.V. (1995). Proline accumulates in plants exposed to UV radiation and protects them against UV-induced peroxidation. Biochem. Biophys. Res. Commun..

[33] Yang S.L., Lan S.S., Gong M. (2009). Hydrogen peroxide-induced proline and metabolic pathway of its accumulation in maize seedlings. J. Plant Physiol..

[34] Domingos P., Prado A.M., Wong A., Gehring C., Feijo J.A. (2015). Nitric oxide: A multitasked signaling gas in plants. Mol. Plant.

[35] Alcázar R., Altabella T., Marco F., Bortolotti C., Reymond M., Koncz C., Carrasco P., Tiburcio A.F. (2010). Polyamines: Molecules with regulatory functions in plant abiotic stress tolerance. Planta.

[36] Vašková J., Kočan L., Vaško L., Perjési P. (2023). Glutathione-related enzymes and proteins: A review. Molecules.

[37] Hasanuzzaman M., Nahar K., Anee T.I., Fujita M. (2017). Glutathione in plants: Biosynthesis and physiological role in environmental stress tolerance. Physiol. Mol. Biol. Pla..

[38] Cassier-Chauvat C., Marceau F., Farci S., Ouchane S., Chauvat F. (2023). The glutathione system: A journey from cyanobacteria to higher eukaryotes. Antioxidants.

[39] Cai J.H., Aharoni A. (2022). Amino acids and their derivatives mediating defense priming and growth tradeoff. Curr. Opin. Plant Biol..

[40] Hildebrandt T.M., Nesi A.N., Araújo W.L., Braun H.P. (2015). Amino acid catabolism in plants. Mol. Plant.

[41] Cao P., Kim S.J., Xing A., Schenck C.A., Liu L., Jiang N., Wang J., Last R.L., Brandizzi F. (2019). Homeostasis of branched-chain amino acids is critical for the activity of TOR signaling in Arabidopsis. Elife.

[42] O’Leary B.M., Oh G.G.K., Lee C.P., Millar A.H. (2020). Metabolite regulatory interactions control plant respiratory metabolism via target of rapamycin (TOR) kinase activation. Plant Cell.

[43] Sheng H., Zhang S., Wei Y., Chen S. (2021). Exogenous application of low-concentration sugar enhances brassinosteroid signaling for skotomorphogenesis by promoting BIN2 degradation. Int. J. Mol. Sci..

[44] Fu L., Wang P., Xiong Y. (2020). Target of rapamycin signaling in plant stress responses. Plant Physiol..

[45] Moreau M., Azzopardi M., Clement G., Dobrenel T., Marchive C., Renne C., Martin-Magniette M.L., Taconnat L., Renou P., Robaglia C. (2012). Mutations in the Arabidopsis homolog of LST8/GβL, a partner of the target of rapamycin kinase, impair plant growth, flowering, and metabolic adaptation to long days. Plant Cell.

[46] Dobrenel T., Caldana C., Hanson J., Robaglia C., Vincentz M., Veit B., Meyer C. (2016). TOR signaling and nutrient sensing. Ann. Rev. Plant Biol..

[47] Li Z., Yang L., Wu Y., Zhang R., Yu S., Fu L. (2024). TOR balances plant growth and cold tolerance by orchestrating amino acid-derived metabolism in tomato. Hortic. Res..

[48] Kumari M., Joshi R., Kumar R. (2020). Metabolic signatures provide novel insights to *Picrorhiza kurroa* adaptation along the altitude in Himalayan region. Metabolomics.

[49] Zhao Y., Xu F., Liu J., Guan F., Quan H., Meng F. (2019). The adaptation strategies of *Herpetospermum pedunculosum* (Ser.) Baill at altitude gradient of the Tibetan plateau by physiological and metabolomic methods. BMC Genom..

[50] Dong X., Guo Y., Xiong C., Sun L. (2020). Evaluation of two major *Rhodiola* species and the systemic changing characteristics of metabolites of *Rhodiola crenulata* in different altitudes by chemical methods combined with UPLC-QqQ-MS-based metabolomics. Molecules.

[51] Papenbrock J., Riemenschneider A., Kamp A., Schulz-Vogt H.N., Schmidt A. (2007). Characterization of cysteine-degrading and H2S-releasing enzymes of higher plants-from the field to the test tube and back. Plant Biol..

[52] Liu H., Wang J., Liu J., Liu T., Xue S. (2021). Hydrogen sulfide (H2S) signaling in plant development and stress responses. Abiotech.

