# Supplementary material for: Transcriptomics and Metabolomics Reveal Mechanisms Underlying the Adaptation of Lamiophlomis rotata to High Altitudes
Source: Biology (Basel). 2025 Nov 5;14(11):1554. doi: 10.3390/biology14111554 (PMC12650051; doi:10.3390/biology14111554)
Supplement: Supplementary file 1 [file biology-14-01554-s001.zip › Supplementary files/Supplementary Figture.pdf]

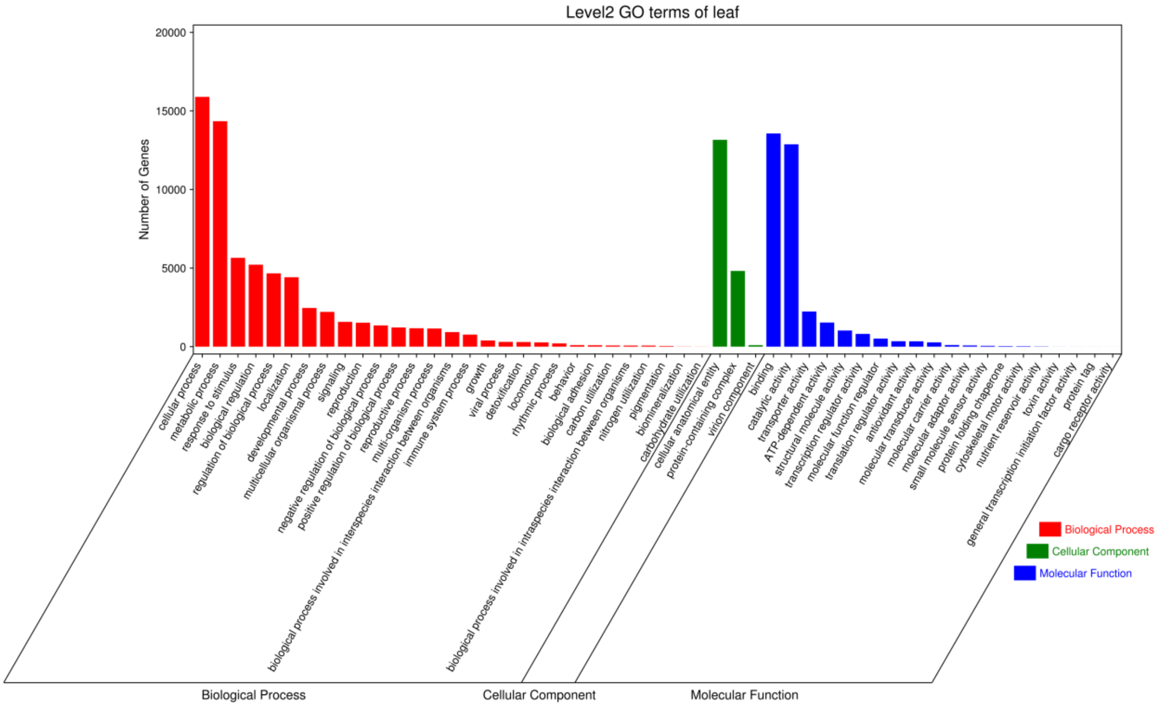

Figure S1 Statistics of GO annotation information

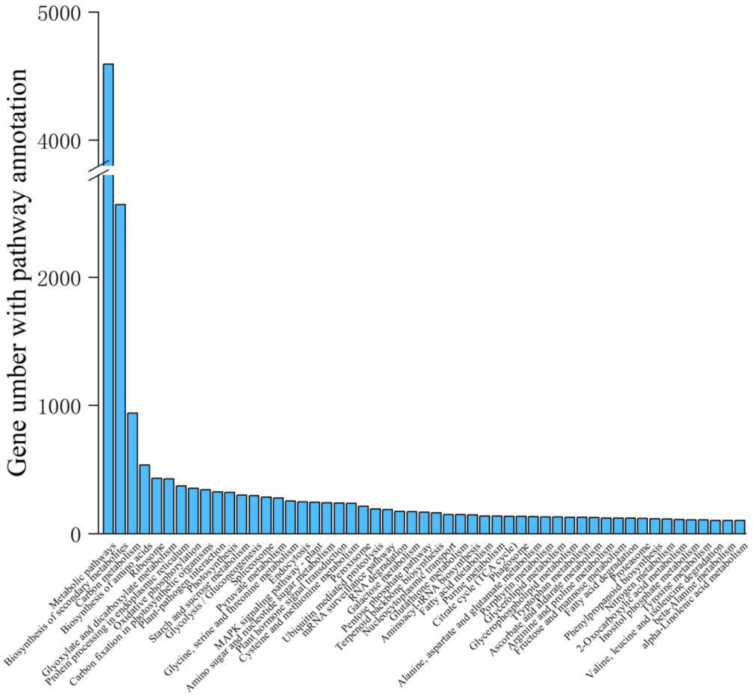

Figure S2 Statistics of KEGG annotation information（Only display the first 53 pathways with the number of gene enrichments greater than 100）

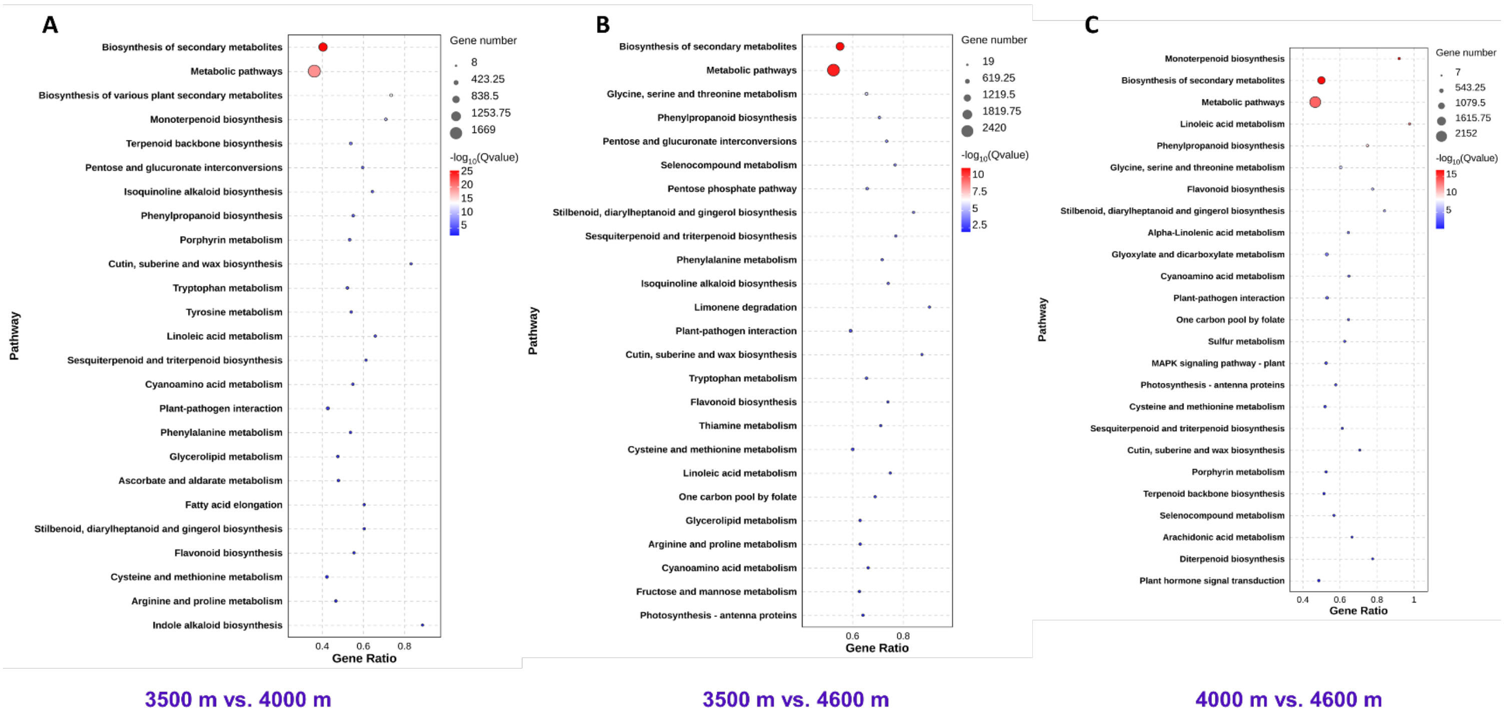

Figure S3 KEGG pathway analysis of DEGs demonstrated that elevated altitude not only activated amino acid metabolism but also modulated the biosynthesis of other metabolites.

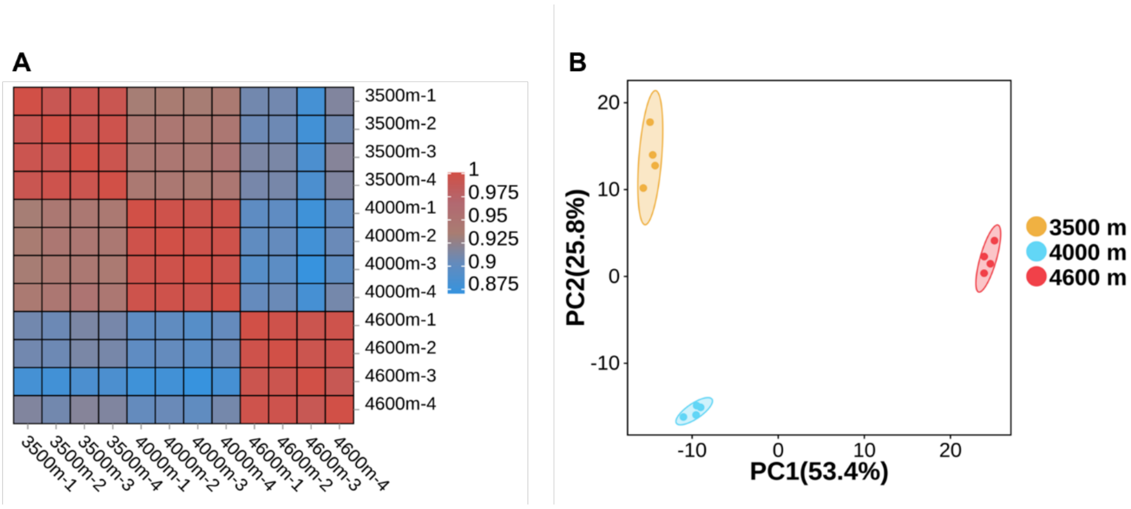

Figure S4 Quality control analysis of metabolomic data. (A)Principal component analysis (PCA) of all the samples. (B) The heatmap of Pearson's correlation of 12 samples.
